# The diagnostic value of narrow-band imaging for early and invasive lung cancer: a meta-analysis

**DOI:** 10.6061/clinics/2017(07)09

**Published:** 2017-07

**Authors:** Juanjuan Zhu, Wei Li, Jihong Zhou, Yuqing Chen, Chenling Zhao, Ting Zhang, Wenjia Peng, Xiaojing Wang

**Affiliations:** I Department of Respiratory Disease, the First Affiliated Hospital of Bengbu Medical College, Bengbu 233004, China; II Department of Epidemiology and Health Statistics, Bengbu Medical College, Bengbu 233030, China; III Provincial Key Laboratory of Respiratory Disease in Anhui, Bengbu 233004, China; IV Department of Respiratory Disease, No.2 People’s Hospital of Fuyang City, Fuyang 236015, China; V Department of Biochemistry and Molecular Biology, Bengbu Medical College, Bengbu 233004, China

**Keywords:** Invasive Lung Cancer, Early Lung Cancer, Narrow-Band Imaging, Meta-Analysis

## Abstract

This study aimed to compare the ability of narrow-band imaging to detect early and invasive lung cancer with that of conventional pathological analysis and white-light bronchoscopy. We searched the PubMed, EMBASE, Sinomed, and China National Knowledge Infrastructure databases for relevant studies. Meta-disc software was used to perform data analysis, meta-regression analysis, sensitivity analysis, and heterogeneity testing, and STATA software was used to determine if publication bias was present, as well as to calculate the relative risks for the sensitivity and specificity of narrow-band imaging *vs* those of white-light bronchoscopy for the detection of early and invasive lung cancer. A random-effects model was used to assess the diagnostic efficacy of the above modalities in cases in which a high degree of between-study heterogeneity was noted with respect to their diagnostic efficacies. The database search identified six studies including 578 patients. The pooled sensitivity and specificity of narrow-band imaging were 86% (95% confidence interval: 83–88%) and 81% (95% confidence interval: 77–84%), respectively, and the pooled sensitivity and specificity of white-light bronchoscopy were 70% (95% confidence interval: 66–74%) and 66% (95% confidence interval: 62–70%), respectively. The pooled relative risks for the sensitivity and specificity of narrow-band imaging *vs* the sensitivity and specificity of white-light bronchoscopy for the detection of early and invasive lung cancer were 1.33 (95% confidence interval: 1.07–1.67) and 1.09 (95% confidence interval: 0.84–1.42), respectively, and sensitivity analysis showed that narrow-band imaging exhibited good diagnostic efficacy with respect to detecting early and invasive lung cancer and that the results of the study were stable. Narrow-band imaging was superior to white light bronchoscopy with respect to detecting early and invasive lung cancer; however, the specificities of the two modalities did not differ significantly.

## INTRODUCTION

Trend analyses of high-quality data from 22 registries, including several population-based registries currently available in the National Central Cancer Registry of China, were conducted in 2000–2011 and published in 2016. These analyses suggested that an estimated 4,292,000 new cancer cases and 2,814,000 cancer deaths would occur in China in 2015, and they identified lung cancer as the most common incident cancer and the leading cause of cancer-related death in the country. Lung cancer is considered an important health threat both in China and in other countries around the world and has imposed significant medical and economic burdens on society 1,2.

Surgery offers a relatively good possibility of a cure in patients with early-stage lung cancer, and the 5-year survival rate for patients with stage Ia disease is 73%. However, the 5-year survival rates range from 9% to 46% in patients with more advanced disease (stages II–IV) 3. Currently, only 16% of lung cancer are diagnosed when the disease is localized, and even fewer lung cancer are diagnosed at stage 0, resulting in an overall 5-year survival rate of only 15%. Therefore, early diagnosis and treatment are important, as these factors are associated with an improved 5-year survival rate and a better prognosis in patients with lung cancer 4.

Endoscopic examination remains the most direct and effective method for detecting lung cancer. Narrow-band imaging (NBI) is a new endoscopic technique designed to detect pathologically altered mucosal and submucosal microvascular patterns that uses the following two narrow light bandwidths: blue light (390–445 nm), which is absorbed by superficial capillaries; and green light (530–550 nm), which is absorbed by blood vessels inside capillary membranes. These narrow bandwidths reduce light scattering (i.e., the deeper the light penetrates into the mucosa, the stronger the scattering) and enable enhanced blood vessel visualization. This approach consequently improves contrast on the mucosal surface, reduces examination times, and eliminates unnecessary biopsies 5.

Following the successful introduction of NBI in the field of gastroenterology and the confirmation of its diagnostic value for gastrointestinal malignancies, clinicians incorporated this modality into interventional pulmonology. NBI has since been used to diagnose lung diseases, especially early and invasive lung cancer. Several studies have compared the ability of NBI to detect early and invasive lung cancer (defined as moderate and severe dysplasia or carcinoma in situ [CIS]) and invasive lung cancer with that of pathological analysis 6-14.

The simplicity, reproducibility, and straightforward nature of NBI evaluations of the tracheo-bronchial tree ensure that NBI videobronchoscopy will play a significant role in lung cancer detection and staging in the future. However, previous publications have shown that the sensitivity and specificity of NBI for these lesions vary widely (0.53–1.00 and 0.43–0.90, respectively). However, most of these studies confirmed that NBI was superior to white-light bronchoscopy (WLB) with respect to detecting both early and invasive lung cancer 7-10,13,14. Therefore, the present study aimed to conduct a meta-analysis to evaluate the diagnostic efficacies of NBI and WLB and to compare the ability of NBI to diagnose early and invasive lung cancer with that of WLB.

## MATERIALS AND METHODS

### Search strategy

We searched the PubMed, EMBASE, Sinomed, and China National Knowledge Infrastructure databases for relevant studies published up to September 14, 2015. We focused on English- and Chinese-language publications reporting the diagnostic efficacy of NBI for early and invasive lung cancer. The following search terms were used to ensure the list of studies retrieved via the search was comprehensive: (“Narrow Band Imaging” OR “Narrowband Imaging” OR “Band Imaging, Narrow” OR “Band Imagings, Narrow” OR “Imaging, Narrow Band” OR “Imagings, Narrow Band” OR “Narrow Band Imagings” OR “Imaging, Narrowband” OR “Imagings, Narrowband” OR “Narrowband Imagings” OR “NBI” OR “Narrow-band Imaging”) AND (“Lungs” OR “Lung” OR “Pulmonary”) AND (“Humans” OR “Human”).

**Figure 1:**
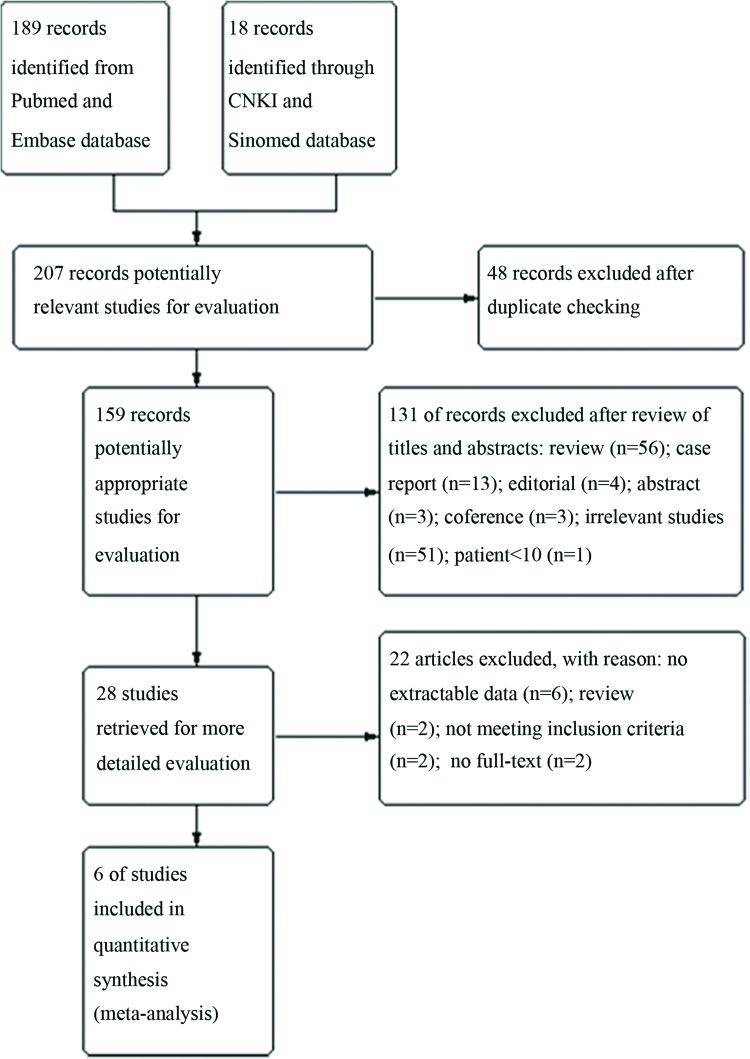
Study flow diagram.

First, all the records from the database search were imported into Endnote to eliminate duplicate studies. Then, two evaluators (WL and JJZ) independently read the titles and abstracts of the articles identified through the above searches to exclude studies that did not meet the analysis inclusion criteria. Any disagreements were resolved through discussion. Finally, the full texts of the remaining studies were carefully reviewed so that informed final decisions regarding their inclusion in the study could be made. The reference lists of the included records were also screened for relevant articles (Figure 1).

### Literature inclusion and exclusion criteria

The following studies were included in the analysis: (a) studies published in English or Chinese; (b) studies using NBI to diagnose early and invasive lung cancer; (c) studies using histopathological analysis as the reference standard (i.e., the gold standard); (d) studies reporting sufficient data for the construction of 2×2 contingency tables for the true positive (TP), false positive (FP), false negative (FN), and true negative (TN) rates of early or invasive lung cancer, as well as studies reporting “per-lesion” or “per-patient” statistics; and (e) studies including patients who underwent clinical or radiological examinations because they were suspected of having or known to have a lung malignancy or were at high risk for lung cancer. When the same data sets or subsets were presented in more than one article, only the article that provided the greatest detail, was published most recently, or had the highest level of quality was chosen. When abstracts and studies did not report sufficient data, we did not attempt to contact their authors to request additional information.

The following studies were excluded from the analysis: (1) studies reporting data pertaining to histologically unconfirmed lesions; (2) studies reporting incomplete data; (3) studies with overlapping data; and (4) letters, editorials, expert opinions, reviews without original data, conference abstracts, meta-analyses, case reports, studies with fewer than 10 cases, and articles for which only the abstract could be obtained.

### Qualitative assessment

Study quality was evaluated using the Quality Assessment of Diagnostic Accuracy Studies (QUADAS) tool 15, a validated tool that assesses diagnostic accuracy studies using 14 domains pertaining to the design and presentation of study data. The possible responses to each question are “yes”, “no”, or “unclear” and are scored as 1, -1, and 0, respectively. The assessment was performed by two independent authors (WL and JJZ), and disagreements were resolved by discussion.

### Data extraction

Data pertaining to the numbers of TP, FP, FN, and TN diagnoses were extracted, and histological pathological findings served as the gold standard with which the above modalities were compared. We constructed 2×2 tables containing the number of cases of early or invasive lung cancer. These data were extracted on both a “per-patient” and a “per-lesion” basis when possible. We also extracted data regarding the first author’s name, year of publication, nation of research origin, mean patient age, ratio of men to women, number of lesions, number of patients, pathological results, number of endoscopists and endoscopes, type of NBI system, study design, and other relevant items (Table 1).

### Data synthesis and analysis

Meta-disc (version 1.4) software was used to test the threshold effect among the included studies by calculating the Spearman correlation coefficient, to combine the indices used to describe the diagnostic efficacy of NBI, and to conduct heterogeneity tests. Study heterogeneity was assessed using the χ^2^ test and I^2^ statistic. I^2^ values of 25%, 50%, and 75% were indicative of low, moderate, and high heterogeneity, respectively. If the I^2^ value exceeded 50%, or the likelihood ratio χ^2^ test yielded a *p*-value <0.05, the data were considered to exhibit significant statistical heterogeneity. Therefore, we pooled the data pertaining to the sensitivity and specificity of the above diagnostic tests using a random-effects model to generate a more conservative estimate.

The area under the summary receiver operating characteristic curve (AUC) was used to analyse the lung cancer diagnostic efficacy of NBI. If significant heterogeneity was noted, meta-regression was used to identify the sources. Subgroup analysis was conducted to examine the diagnostic value of various statistical indices, and sensitivity analysis was used to assess the stability of the study results.

STATA software (version 11.0; STATACorp, College Station, TX, USA) was used to calculate the relative risks (RRs) for the sensitivity and specificity of NBI vs the sensitivity and specificity of WLB for the diagnosis of early or invasive lung cancer. For these analyses, a *p-*value <0.05 was considered significant. An RR >1 indicated that NBI was more sensitive than WLB for the detection of early invasive lung cancer. Publication bias was analysed using a weighted linear regression model and a Deeks funnel plot (STATA software). An asymmetric funnel plot was suggestive of possible publication bias. The significance of the intercept was determined using a t test, and a *p-*value <0.05 was indicative of statistically significant publication bias. All *p*-values were two-sided, and all confidence intervals (CIs) had a two-sided probability coverage of 95%.

## RESULTS

### Study identification and study quality

Of the 207 reports retrieved during the database search, 159 remained after the duplicate studies were eliminated, and 28 remained after the titles and abstracts were screened. Twenty-two studies were excluded after a full-text review for the following reasons: (a) their biopsies were performed at sites of abnormal fluorescence, (b) they reported insufficient data with respect to their sensitivity and specificity calculations, (c) they were review articles, (d) their data were presented in more than one article, and (e) their full text was not available (Figure 1).

### Quality assessment

The results of the quality assessment of the eligible studies are shown in Table 1. The studies included in the analysis were generally of an ideal design.

### NBI accuracy

**Figure 2:**
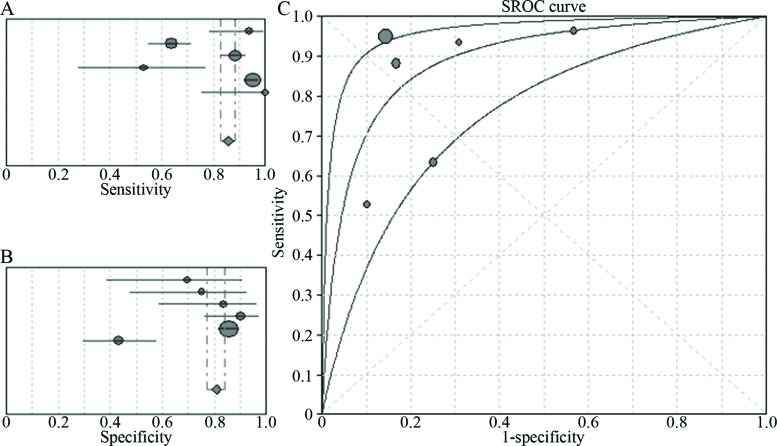
Six-study analysis of the ability of NBI to diagnose early lung cancer and invasive lung cancer. **A.** Pooled sensitivity of NBI for the diagnosis of early lung cancer and invasive lung cancer; **B.** Pooled specificity of NBI for the diagnosis of early lung cancer and invasive lung cancer; **C.** The summary receiver operating characteristic (SROC) curve for the diagnosis of early lung cancer and invasive lung cancer by NBI.

Five prospective studies and one retrospective study including a total of 578 patients were included in the analysis. The Spearman correlation coefficient for these studies was 0.600, *p*=0.208, indicating that the lack of a definite threshold effect produced heterogeneity. The I^2^ values for sensitivity and specificity were 94.1% and 88.9%, respectively, indicating that a high level of between-study heterogeneity was present with respect these parameters because of the non-threshold effect. When a random-effects model was used to analyse the sensitivity and specificity of NBI for the detection of early or invasive lung cancer, the pooled effect values for the sensitivity, specificity, and AUC were 86% (95% CI: 83–88%, I^2^=94.1%), 81% (95% CI: 77–84%, I^2^=88.9%), and 0.89 (Figure 2).

### The efficacies of NBI and WLB

**Figure 3:**
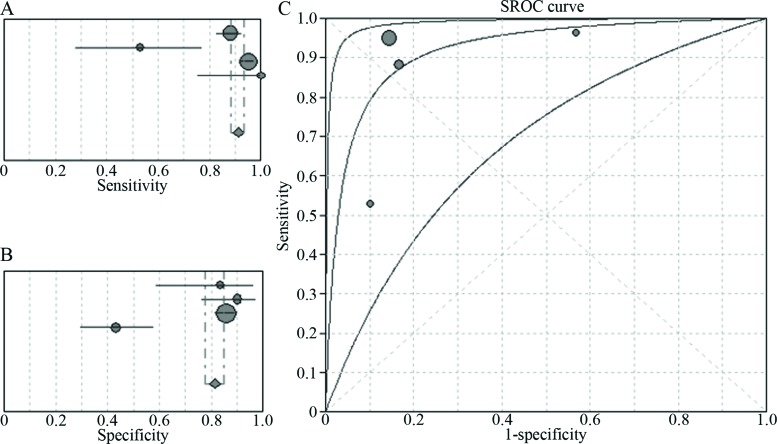
Four-study analysis of the ability of NBI to diagnose early lung cancer and invasive lung cancer. **A.** Pooled sensitivity of NBI for the diagnosis of early lung cancer and invasive lung cancer; **B.** Pooled specificity of NBI for the diagnosis of early lung cancer and invasive lung cancer; **C.** The SROC curve for the diagnosis of early lung cancer and invasive lung cancer by NBI.

**Figure 4:**
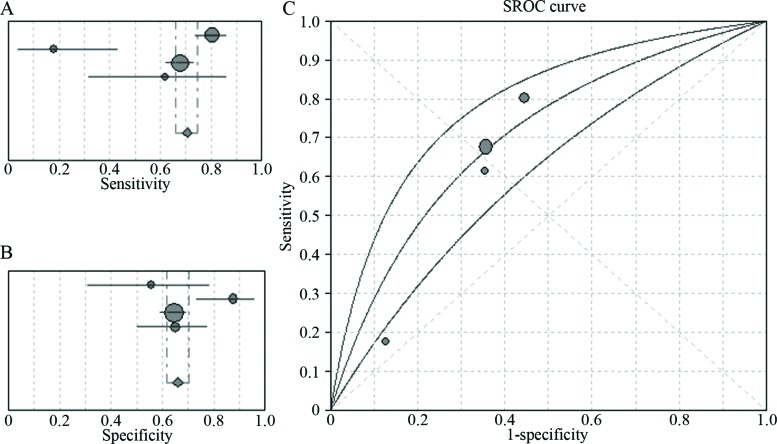
Four-study analysis of the ability of WLB to diagnose early lung cancer and invasive lung cancer. **A.** Pooled sensitivity of WLB for the diagnosis of early lung cancer and invasive lung cancer; **B.** Pooled specificity of WLB for the diagnosis of early lung cancer and invasive lung cancer; **C.** The SROC curve for the diagnosis of early lung cancer and invasive lung cancer by WLB.

**Figure 5:**
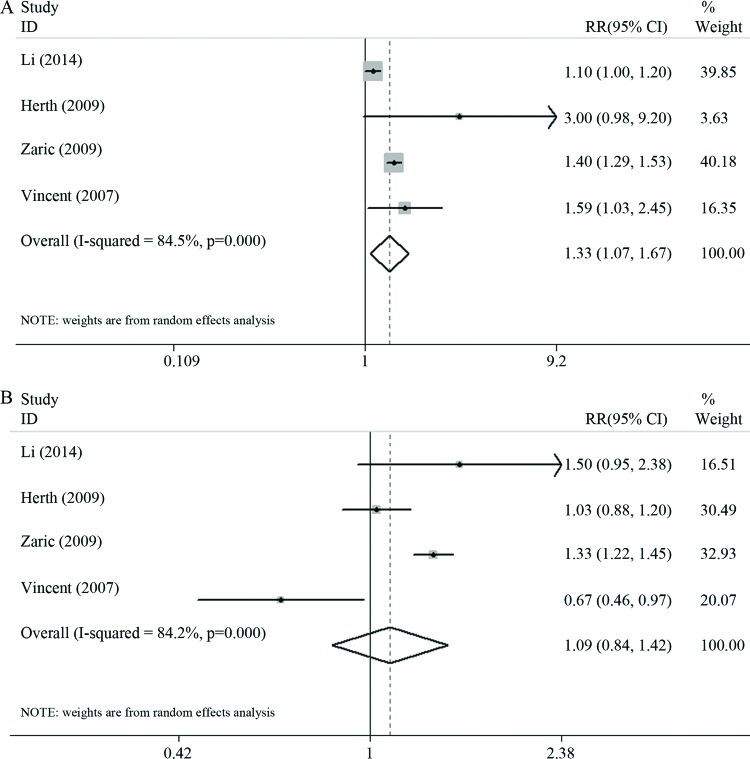
**A.** The pooled RR for the sensitivity of NBI vs the sensitivity of WLB for the detection of early lung cancer and invasive lung cancer; **B.** The pooled RR for the specificity of NBI vs the specificity of WLB for the detection of early lung cancer and invasive lung cancer.

Four of the included articles 7-10 compared the diagnostic efficacies of NBI and WLB, and three of these articles included data pertaining to patients with of moderate and severe dysplasia. These four studies included a total of 381 patients and 1870 lesions. The pooled sensitivity and specificity of NBI for the detection of early and invasive lung cancer were 91% (95% CI: 88–94%, I^2^=89%) and 81% (95% CI: 77–85%, I^2^=93.1%), respectively, with an AUC of 0.92 (Figure 3). The pooled sensitivity and specificity of WLB for the detection of early and invasive lung cancer were 70% (95% CI: 66–74%, I^2^=90.3%) and 66% (95% CI: 0.62–0.70, I^2^=72.8%), respectively, with an AUC of 0.70 (Figure 4). The pooled RR for the sensitivity of NBI *vs* the sensitivity of WLB for the detection of early and invasive cancer was 1.33 (95% CI: 1.07–1.67), and the corresponding pooled RR for the specificity of NBI vs the specificity of WLB for the detection of early or invasive cancer was 1.09 (95% CI: 0.84–1.42) (Figure 5). Thus, NBI was significantly more sensitive than WLB with respect to detecting early and invasive lung cancer; however, NBI and WLB did not differ significantly with respect to specificity.

#### The value of NBI *vs* that of WLB for the diagnosis of early lung cancer

We also searched for data regarding the ability of NBI to accurately diagnose early lung cancer only 7,8. The sensitivity and specificity of NBI for the diagnosis of early lung cancer were 62% (95% CI: 38–82%, I^2^=77.3%) and 58% (95% CI: 48–68%, I^2^=96.8%), respectively, whereas the corresponding values for WLB were 14% (95% CI: 3–36%, I^2^=27.6%) and 69% (95% CI: 59–78%, I^2^=91.4%), respectively. The RR for the sensitivity of NBI vs the sensitivity of WLB for the diagnosis of early lung cancer was 3.86 (95% CI: 1.38–10.75), and the RR for the specificity of NBI vs the specificity of WLB for the diagnosis of early lung cancer was 0.83 (95% CI: 0.45–1.53). Therefore, we concluded that NBI is superior to WLB with respect to detecting early lung cancer. The sensitivity and specificity of NBI were lower in the above studies than in the cumulative results.

#### The value of NBI *vs* that of autofluorescence imaging and NBI + autofluorescence imaging

In this study, we also searched for data comparing the ability of NBI to accurately diagnose early and invasive lung cancer with that of autofluorescence imaging (AFI) and NBI + AFI 7,11. Only two studies including 210 patients were included in this analysis, and we applied a fixed-effects model to pool the effect sizes. The diagnostic sensitivity and specificity of NBI for the diagnosis of early and invasive lung cancer were 62% (95% CI: 54–70%, I^2^=0.0%) and 86% (95% CI: 0.74–0.94, I^2^=48.2%), respectively, whereas the corresponding values of AFI were 91% (95% CI: 85–95%, I^2^=90.7%) and 38% (95% CI: 25–51%, I^2^=0.0%), respectively. The RRs for the sensitivity and specificity of NBI for the diagnosis of early and invasive lung cancer were 0.69 (95% CI: 0.60–0.78) and 2.29 (95% CI: 1.61–3.25), respectively.

The sensitivity and specificity of AFI + NBI for the detection of early and invasive lung cancer were 93% (95% CI: 88–96%, I^2^=89.3%) and 54% (95% CI: 40–67%, I^2^=91.3%), respectively, and the corresponding RRs were 0.67 (95% CI: 0.59–0.76) and 1.38 (95% CI: 0.49–3.90), respectively. Therefore, we concluded that the sensitivity of NBI for the diagnosis of early and invasive lung cancer was lower than that of AFI + NBI; however the specificity of NBI for the diagnosis of early and invasive lung cancer did not differ significantly from that of AFI, suggesting that NBI and AFI are complementary tests. Unfortunately, only two relevant studies regarding the ability of these tests to diagnosis early and invasive lung cancer were available; thus, additional studies will be needed to confirm the abovementioned findings.

### Sensitivity and meta-regression analysis

As shown in Table 2, we noted high heterogeneity in the pooled effect sizes from the included studies. Therefore, we performed sensitivity analysis of the ability of NBI to diagnose early and invasive lung cancer using characteristics from different types of studies, including prospective studies and studies including ≥50 patients and using per-patient statistics and different video endoscopy systems or patient populations, as well as other studies (Table 2). In addition, we sequentially excluded studies whose results deviated significantly from the pooled effect size 7,8. The remaining five and four studies were subjected to sensitivity analyses. The AUCs of NBI for the diagnosis of early and invasive cancer ranged from 0.83 to 0.93. These results indicated that the diagnostic efficacy of NBI for early and invasive cancer remained high and that the results of the study were stable.

Several of the previously mentioned factors were also selected for the meta-regression analysis (Table 3). Although all the factors could be extracted from the six studies, the sources of between-study heterogeneity were not identified. Notably, the heterogeneity may have been caused by factors that could not be assessed in the present analysis, namely, patient age and sex ratios, because a portion of their data were lost or were not reported.

### Assessment of publication bias

**Figure 6:**
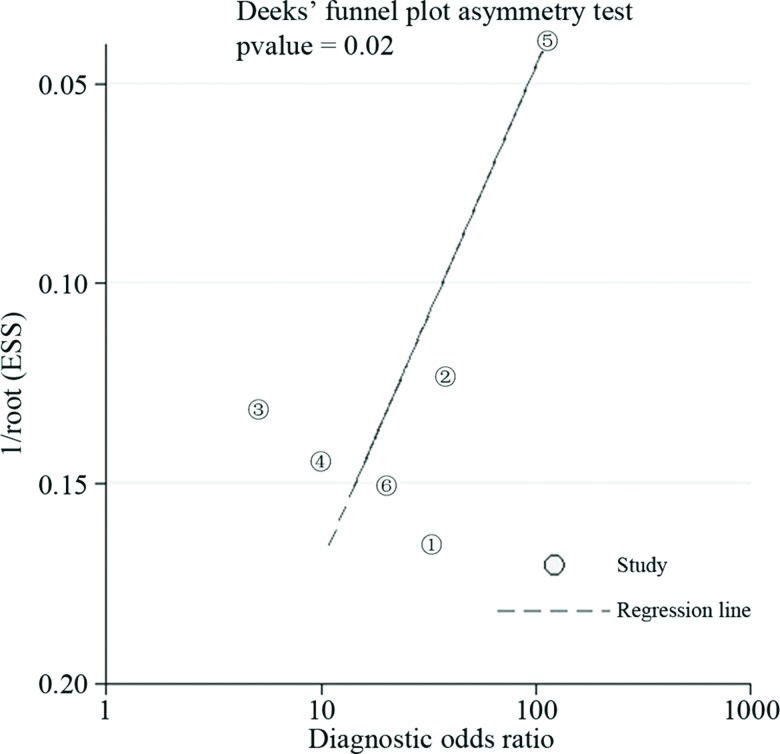
Deeks funnel plot for the evaluation of publication bias.

In the publication bias analysis (STATA), the Deeks funnel plot suggested that publication bias may have been present among the included studies, and the corresponding *p-*value of 0.02 indicated that statistically significant publication bias was present among the included studies. As shown in the Deeks funnel plot (Figure 6), the angle of the regression line and the diagnostic odds ratio axis diverged from 90°, suggesting that publication bias was present among the included studies.

### Complications

Two studies reported adverse effects. In one study, all the adverse effects were attributed to bronchoscopy rather than to the NBI devices. The other study reported slight bleeding that was attributed to NBI but described no other NBI-related complications.

## DISCUSSION

Diagnosing lung cancer early is essential for effectively treating the disease and optimizing survival. Bronchoscopy is ideal for detecting lung cancer arising from the central airway; however, detecting early and invasive lung cancer and precancerous lesions using WLB remains difficult. According to a previous report, only 29% of CIS and 69% of microinvasive cancer cases are identified by WLB, regardless of the experience level of the bronchoscopist 16. The more advanced AFI bronchoscopy exploits the autofluorescent nature of the bronchial mucosa to detect tiny and subtle superficial lesions. AFI has been shown to increase the yield of diagnostic testing for early lung cancer 17-22; however, its role in detecting lung neoplasia is limited by its specificity, which is actually lower than that of WLB and leads to more false positives 7,21,23,24.

NBI is an optical technique based on the use of narrower blue and green light filters to enhance visualization of the microvascular architecture and microsurface structures. This technique, which offers chromoendoscopy without requiring additional dye sprays, clearly highlights the micromorphology and microvascular structures of mucosal lesions and facilitates the identification of cancerous lesions 5,25-27. NBI is simple and can be performed by general endoscopists; however, it is also an accepted endoscopic diagnostic method with defined standards for the diagnosis of early and invasive lung cancer. The most widely used set of current diagnostic standards was proposed by Shibuya, whose “descriptors” include dotted, tortuous, abruptly terminating blood vessels 27.

NBI can distinguish cancer from benign lesions with a high level of accuracy 6,25,26. Several articles have reported the superiority of NBI over AFI and WLB because the former detects cancerous lesions with higher specificity than the latter; however its sensitivity is not significantly compromised by its higher specificity 7-11,13,14. In a meta-analysis of 14 studies (15 data sets), Chen et al. 23 compared the ability of autofluorescence bronchoscopy (AFB) to detect lung cancer and paraneoplastic lesions with that of WLB. The pooled sensitivity and specificity for AFB were 90% (95% CI: 84–93%) and 56% (95% CI: 45–66%), respectively, and the pooled sensitivity and specificity for WLB were 66% (95% CI: 58–73%) and 69% (95% CI: 57–79%), respectively. Wang et al. 21 also compared the ability of AFB to detect lung cancer and precancerous lesions with that of WLB in a meta-analysis of six studies. The pooled sensitivities of AFI and WLB were 89% (95% CI: 81–94%) and 67% (95% CI: 46–83%), respectively, and the pooled specificities of AFI and WLB were 64% (95% CI: 37–84%) and 84% (95% CI: 74–91%), respectively. Thus, AFI was more sensitive and less specific than WLB for the detection for early and invasive lung cancer. However, in the present meta-analysis, NBI had a sensitivity of 86% (95% CI: 83–88%, I^2^=94.1%) and a specificity 81% (95% CI: 77–84%, I^2^=88.9%) for the detection of early and invasive lung cancer.

One systematic review 28 from 2001 that examined the utility of positron emission tomography (PET) with respect to the diagnosis of pulmonary nodules and mass lesions included 40 studies that met its specific inclusion criteria. PET was used to examine 1474 focal pulmonary lesions of any size and was found to have a maximum joint sensitivity and specificity (upper-left point on the receiver operating characteristic curve at which sensitivity and specificity were equal) of 91.2% (95% CI: 89.1–92.9%). The diagnostic sensitivity and specificity of PET are superior to those of NBI; however, PET is expensive, cannot be used to obtain histological specimens, and exposes the patient to radiation.

The prognoses of patients with lung cancer depend heavily on the stage at which patients are diagnosed with the disease. According to the stepwise progression theory of carcinogenesis, early detection of preinvasive lesions, such as moderate or severe dysplasia or CIS, and subsequent prompt surgical resection or endobronchial treatment will provide the patient with the best chance of survival and lung function conservation. Consistent with this idea, the reported 5-year survival rate for patients treated for preinvasive (stage 0) lung cancer exceeds 90% 29. However, in the current study, only two articles 7,8 evaluated the ability of NBI and WLB to accurately diagnose early lung cancer. The sensitivity and specificity of NBI for the diagnoses of early lung cancer were 62% and 58%, respectively, and the corresponding values for WLB were 14% and 69%, respectively. The corresponding RRs for the sensitivity and specificity were 3.86 (95% CI: 1.38–10.75) and 0.83 (95% CI: 0.45–1.53), respectively. Thus, NBI appears to be superior to WLB with respect to detecting early lung cancer. Unfortunately, only two relevant studies regarding this issue were identified, and additional research is needed to confirm the findings described herein.

NBI, a novel observational method, has facilitated pathological diagnoses of lung cancer by allowing mass observation, guiding targeted biopsies, and enabling predictions of pathological type 30; has improved assessments of extension; and has influenced therapeutic strategies 9,10,14. The results of our meta-analysis suggest that NBI is highly accurate with respect to diagnosing lung neoplasms.

An article 7 included in the analysis had accepted patients who were at high risk for lung cancer (i.e., patients without a known diagnosis or clinical findings suggestive of central airway malignancies). In that study, a histologic tumour grade of moderate to severe dysplasia or CIS (via biopsy) was considered a positive diagnosis of intraepithelial neoplasia. However, that study reported a lower NBI sensitivity for the detection of lung cancer than other recent articles 13,18. This discrepancy can be explained by the ability of endoscopy to distinguish between “abnormal” and “suspicious” lesions and the researchers’ subsequent decision to consider only suspicious lesions to be positive diagnostic findings on bronchoscopy. Not all previous studies made this distinction, and if that study had considered both abnormal and suspicious lesions to be “positive bronchoscopic findings”, NBI would have identified 100% of the high-grade dysplastic lesions and CIS affecting the patients enrolled therein. Similarly, Vincent et al. 8 reported that NBI identified dysplasias or malignancies that were not detected by WLB in 23% of patients. However, NBI did not detect these lesions with greater accuracy in areas with abnormal WLB findings. Further studies are needed to determine the efficacy of NBI with respect to the detection of premalignant airway lesions in at-risk populations.

In an earlier article, Herth reported that AFI, NBI, and NBI + AFI exhibited sensitivities of 65%, 53%, and 71% and specificities of 40%, 90%, and 40%, respectively, for the diagnosis of early lung cancer 7. Although NBI was significantly more specific than AFI with respect to diagnosing cancer (*p*<0.001), these modalities did not differ significantly with regards to sensitivity (*p*=0.49). Furthermore, although the relative sensitivity for the combination of AFI and NBI *vs* WLB was 4.0, the combined test did not exhibit improved specificity compared with either AFI alone or NBI alone (*p*=0.56). The specificity of AFI + NBI was equal to that of AFI and significantly lower than that of NBI; thus, this combination led to a nonsignificant increase in lung cancer diagnostic yield. In summary, this study demonstrated that NBI can be used alone as a first-choice lung cancer diagnostic modality rather than in combination with other diagnostic modalities.

According to Chen 11, the diagnostic sensitivities of NBI, AFI, and NBI + AFI for the detection of central lung cancer were 63.5%, 94.2%, and 95.6%, respectively, and the specificities of these tests for the detection of central lung cancer were 75.0%, 31.3%, and 87.5%, respectively. The specificity and sensitivity of NBI were significantly higher and lower than those of AFI, respectively, suggesting that NBI and AFI are complementary tests. In that study, NBI + AFB and AFB alone differed significantly with respect to their specificities (*p*<0.01) but not their sensitivities (*p*>0.05) for the diagnosis of early and invasive lung cancer, whereas NBI + AFB and NBI alone differed significantly with regards to both their sensitivities and their specificities for the diagnosis of early and invasive lung cancer (*p*<0.01 and *p*=0.03, respectively). Chen concluded that NBI + AFB could diagnose lung cancer with greater specificity than AFB alone, a notable finding that contradicted those reported by Herth. This discrepancy may be attributable to the following factors: 1. The study by Chen addressed obvious bronchial and pulmonary masses and used the above diagnostic techniques only to observe mucosal abnormalities. Moreover, the patients eligible for inclusion in the study were not at high risk for lung cancer. Therefore, that study differed from the study performed by Herth, as it lacked data regarding certain study parameters, as well as data regarding precancerous lesions. 2. The positive diagnosis rate reported by Chen pertained to patients rather than lesions. The discrepancies between these two studies have necessitated additional high quality research to determine whether NBI + AFB is superior to either modality alone with respect to diagnosing cancer.

Despite these differences, the study by Chen demonstrated the distinct advantages of the above modalities. AFB is used to observe surface mucosal changes, and NBI is used to observe surface mucosal vascular lesions. When used in combination, each modality can compensate for the shortcomings of the other. However, AFI and NBI are not currently integrated into single bronchoscope units. In the future, the combined use of these technologies may reduce inspection times and patient discomfort. In our meta-analysis, which included 578 patients, only one article reported slight bleeding. Therefore, we believe that NBI, which has high diagnostic value and a low complication rate, may be an accurate, safe, and cost-effective tool for diagnosing early lung and invasive lung cancer.

### Limitations

Our analysis had some limitations. First, we included only six studies in the analysis. Second, we were unable to identify the sources of heterogeneity among the studies included herein. Although one could argue that heterogeneity is inevitable in a meta-analysis regardless of whether its presence is detectable via statistical analysis, its existence limits the clinical application of the results of the analysis. The heterogeneity noted herein may be attributed to differences in the time spans of the included studies, the use of different types of bronchoscopes, differences in the experience levels of the endoscopists and pathologists who performed the bronchoscopies and biopsies, diagnostic research on small sample sizes, differences in sex ratios and age distributions, and differences in pathological results. Furthermore, in some of the studies included in the analysis, tissue samples biopsied from visually normal areas, as demonstrated by both NBI and WLB, were used as controls; however, other studies included in the analysis did not perform control biopsies. Third, in our subgroup analysis, we compared the diagnostic accuracies of NBI, AFI and AFI + NBI; however, we identified only two studies that performed these comparisons. Therefore, our conclusions regarding the relative efficacies of those modalities require further verification in future studies. Fourth, all the included studies used an earlier bronchoscope model. The newest NBI bronchoscope is the OLYMPUS BF-H290, which produces clearer images than earlier models but is expensive. To date, no published studies have used this bronchoscope. Finally, publication bias was inevitable because we did not search for unpublished data, and data regarding TP, FP, TN, and FN diagnoses could not be extracted from the sensitivity and specificity calculations of two relevant studies 13,14. In addition, we included only English- and Chinese-language studies from four major databases and therefore may have overlooked some important articles published in other countries and languages. Furthermore, the reports included herein seldom described whether their patients were consecutively or randomly selected, an omission that may also have been a source of bias.

NBI is an accurate, safe, and cost-effective tool for diagnosing lung cancer. The current evidence indicates that NBI is more valuable than WLB with respect to diagnosing both early and invasive lung cancer and that it is both feasible and convenient to switch between these two modalities as technologies advance. Our findings indicate that NBI is superior to WLB and that its use may lead to improvements in diagnostic yields, as well as improvements in sensitivity for the diagnosis of cancer that do not compromise specificity. However, additional high-quality prospective studies regarding the ability of NBI to diagnose early and invasive lung cancer remain necessary.

## AUTHOR CONTRIBUTIONS

Li W designed the study, acquired some of the data, edited the manuscript and critically reviewed the final version of the manuscript. Zhu J acquired some of the data, conducted the statistical analysis, and drafted the manuscript. Chen Y, Zhao C, Zhang T, Peng W, Wang X performed the study and performed the data collection and analysis. All authors read and approved the final version of the manuscript.

## Figures and Tables

**Table 1:** Characteristics of the studies included in this meta-analysis

Study/year	Nation	No. of subjects (n)	No. of biopsies (n)	Average age (yr)	M/F	Control	Statistical index	Blind	QUADAS score
Gao et al./2014 12	China	44	Unclear	58.3±13.8	34/10	No	Per-patient	Unclear	11
Li et al./2014 9	China	196	1072	58±10	152/44	No	Per-patient	Yes	12
Chen et al./2014 11	China	153	Unclear	57±11	106/47	No	Per-patient	Yes (partially blind)	12
Herth et al./2009 7	Germany	57	98	Unclear	Unclear	No	Per-patient	Yes	11
Bojan et al./2009 10	Serbia	106	636	55	85/21	Yes	Per-lesion	Yes	13
Vincent et al./2007 8	America	22	64	59	8/14	Yes	Per-lesion	Yes (partially blind)	11

**Table 1:** Characteristics of the studies included in this meta-analysis (continued)

Study/year	Type of investigation	NBI System	No. of endoscopists	Lesion characterization	Study design	Pathological results
Gao et al./2014 12	NBI	EVIS LUCERA (CV-260SL)	2	Shibuya’s descriptors	Retrospective	Cancer
Li et al./2014 9	NBI, WLB	EVIS LUCERA (CV-260SL)	Unclear	Shibuya’s descriptors	Prospective	Moderate to severe dysplasia and Cancer
Chen et al./2014 11	NBI, AFI+NBI, AFI	EVIS LUCERA (CV-260SL)	Unclear	Shibuya’s descriptors	Prospective	Cancer
Herth et al./2009 7	NBI, AFI, WLB and combination	EVIS EXERA	Unclear (more than one)	Shibuya’s descriptors	Prospective	Moderate to severe dysplasia and carcinoma in situ
Bojan et al./2009 10	NBI, WLB	EVIS LUCERA (CV-260SL)	Unclear	Shibuya’s descriptors	Prospective	Cancer
Vincent et al./2007 8	NBI, WLB	EVIS EXERA II	2	Shibuya’s descriptors	Prospective	Moderate to severe dysplasia and cancer

**Table 1:** Characteristics of studies included in this meta-analysis (continued)

Study/year	No. of subjects (n)	TP	FP	FN	TN
Gao et al./2014 12	44	29	4	2	9
Li et al./2014 9	196	157	3	21	15
Chen et al./2014 11	153	87	4	50	12
Herth et al./2009 7	57	9	4	8	36
Bojan et al./2009 10	106	267	51	14	304
Vincent et al./2007 8	22	13	29	0	22

**Table 2:** Outcomes of the sensitivity analysis of selected studies assessing the stability of the accuracy of NBI for diagnosing early and invasive lung cancer

Study characteristics	No. of studies	No. of patients	Pooled estimates^d^ Sensitivity Specificity		AUC
Inclusion population^a^ 8-12	5	521	0.86 (0.84-0.89)	0.80 (0.76-0.84)	0.89
Prospective studies 8-11	5	534	0.85 (0.82-0.88)	0.81 (0.77-0.84)	0.89
Studies with ≥50 patients 7,9-11	4	512	0.85 (0.82-0.88)	0.86 (0.82-0.89)	0.91
EVIS LUCERA videoendoscopy system 9-12	4	499	0.86 (0.83-0.89)	0.85 (0.81-0.88)	0.87
Final outcome^b^ 10-12	3	303	0.86 (0.81-0.90)	0.67 (0.57-0.76)	0.89
Per-patient analysis 7, 9, 11, 12	4	450	0.78 (0.73-0.82)	0.83 (0.73-0.90)	0.87
Blind 7-11	5	534	0.85 (0.82-0.88)	0.81 (0.77-0.84)	0.89
No control 7,9,11,12	4	450	0.78 (0.73-0.82)	0.83 (0.73-0.90)	0.87
Five studies^c^ 7,9-12	5	556	0.85 (0.82-0.88)	0.85 (0.81-0.88)	0.90
Four studies^c^ 9-12	4	499	0.86 (0.83-0.89)	0.85 (0.81-0.88)	0.87

a Patients with known or suspected lung malignant lesions.

b Pathological positive index was cancer.

c Studies whose results deviated significantly from the pooled effect size were excluded, and sensitivity analyses of the remaining five and four studies were performed.

d The 95% confidence interval is given in parentheses.

**Table 3:** Meta-regression analysis of the potential sources of heterogeneity

Variables	Coefficient	Relative DOR (95% CI)	*p*-value
Study design (prospective vs retrospective)	0.311	1.36 (0.01-285.42)	0.8650
Number of patients (≥50 vs <50)	1.768	5.86 (0.05-750.93)	0.3303
Histology outcome (invasive cancer vs early lung cancer)	-0.139	0.87 (0.02-33.32)	0.9111
Videoendoscopy system (Lucera vs EXERA)	-0.604	0.55 (0.01-32.01)	0.6691
Statistical index (per-patient vs per-lesion)	1.400	4.06 (0.14-118.82)	0.2787
Included population (known or suspected lung malignancy vs high risk of lung malignancy)	-0.083	0.92 (0.00-801.28)	0.9712
